# Influence of Pain on Knee Joint Movement and Moment during the Stance Phase in Patients with Severe Bilateral Knee Osteoarthritis: A Pilot Study

**DOI:** 10.3390/medicina55120756

**Published:** 2019-11-22

**Authors:** Takashi Fukaya, Hirotaka Mutsuzaki, Koichi Mori

**Affiliations:** 1 Department of Physical Therapy, Faculty of Health Sciences, Tsukuba International University, 6-8-33 Manabe, Tsuchiura, Ibaraki 300-0051, Japan; 2 Center for Medical Sciences, Ibaraki Prefectural University of Health Sciences, 4669-2 Ami-machi, Inashiki, Ibaraki 300-0394, Japan; mutsuzaki@ipu.ac.jp; 3 Department of Orthopaedic Surgery, Ibaraki Prefectural University of Health Sciences Hospital, 4773 Ami-machi, Inashiki, Ibaraki 300-0394, Japan; 4 Department of Radiological Sciences, Ibaraki Prefectural University of Health Sciences, 4669-2 Ami-machi, Inashiki, Ibaraki 300-0394, Japan; mori@ipu.ac.jp

**Keywords:** osteoarthritis, knee, movement, pain

## Abstract

*Background and Objectives:* The purpose of this study was to compare the side-to-side differences in knee joint movement and moment for the degree of pain in the walking stance phase in patients with bilateral knee osteoarthritis (KOA) of comparable severity. We hypothesized that knee joint movement and moment on the side with strong pain were lower compared with the side with weak pain. *Materials and Methods:* We included 11 patients diagnosed with bilateral severe KOA. In all patients’ left and right knees, the Kellgren–Lawrence radiographic scoring system grade was level 4, and the femorotibial angle and knee range of motion were equivalent. Following patients’ interviews with an orthopedic surgeon, we performed a comparative study with KOA with strong pain (KOAs) as the strong painful side and KOA with weak pain (KOAw) as the weak painful side. Data for changes in bilateral knee joint angles in three dimensions during the stance phase and bilateral knee sagittal and frontal moments exerted in the early and late stance phases were extracted from kinematics and kinetics analyses. *Results:* Three-dimensional joint movements in the knee joint were not significantly different in all phases between KOAs and KOAw. Knee extensor moment in the early stance phase in KOAs was significantly smaller than that in KOAw. Knee abductor moment in the early and late stance phase was not significantly different between KOAs and KOAw. *Conclusions:* Although we found no difference in joint motion in bilateral knee joints, knee extensor moment on the side with strong pain was decreased. In patients with bilateral severe KOA, it was suggested that the magnitude of knee pain contributed to the decrease in knee joint function.

## 1. Introduction

Knee osteoarthritis (KOA) is associated with advancing age and biomechanical and biological changes in the knee joint. In Japan, radiographic KOA is observed in approximately 40% of men and 60% of women >40 years of age [1]. KOA progression is accompanied by impaired mechanical function of the knee joint during weight-bearing activities and nonconformity of the knee joint surface created by articular cartilage wear [2]. KOA involves stiffness of joint movement accompanied by joint structure failure in the stance phase of walking [3] and decreased internal knee extensor moment secondary to muscle weakness in the knee joint extensor muscles by the quadriceps muscle [4]. The main symptom is pain, which is the most common complaint, and which causes functional limitation [5]. Several previous reports stated that patients with KOA have altered kinematics and kinetic patterns in their lower limbs during walking, to avoid pain and joint load. For example, decreased knee flexion angle [6], gait velocity [7], toe-out gait [8], and trunk lean [9] were reported.

Clinically, KOA often progresses, and both sides can be highly and severely affected. If the KOA is severe in bilateral knee joints, further muscle weakness and range of motion limitation occurs, and the limitations in activities of daily living become remarkable. However, in patients with KOA of equal severity in bilateral knee joints, the effect of side-to-side differences in knee joint kinematics and kinetics during walking on the degree of pain is not fully understood. Even with similar bilateral severe KOA, treatment and rehabilitation strategies may need to change if there are differences in knee function, depending on the degree of pain. In this study, we hypothesized that knee joint movement and moment on the side with strong pain were lower compared with the side with weak pain.

The purpose of this study was to compare differences in knee joint movement and moment in the walking stance phase in patients with KOA of comparable severity in each knee, and to clarify the side-to-side differences in knee joint function associated with the degree of pain.

## 2. Materials and Methods

### 2.1. Patients

This study was conducted at the Ibaraki Prefectural University from 2015 to 2019 and patients were selected consecutively. We included 11 patients diagnosed with KOA with grade 4 level Kellgren–Lawrence radiographic scores [10] in bilateral knee joints who were scheduled to undergo total knee arthroplasty in one knee joint. All patients did not receive physical therapy prior to measurement. Internal medication continued until preoperatively, but joint injection and poultice were discontinued 1 month before the operation. Patients’ characteristics are shown in Table 1. In patients’ left and right knees, the femoro-tibial angle (FTA) and knee range of motion (ROM) were equivalent. The knee pain by numerical rating scale (NRS) showed a significant difference (t (10) = 12.0, *p* < 0.001, d = 0.55). As a result of the pain evaluation, all the subjects showed higher results of 1 to 2 points on the knee joint on the side that was facing the operation. Following patients’ interviews with an orthopedic surgeon, we performed a comparative study with KOAs as the strong painful knee (KOAs) and as the weak painful knee (KOAw).

The exclusion criteria were prior knee replacement surgery, or unresolved injuries to any joint of the lower extremities and prior brain injury or other central nervous system diseases under treatment. All participants provided written informed consent, and the study was performed according to the guidelines of the Declaration of Helsinki. Ethical approval for this study was obtained from the Ibaraki Prefectural University of Health Sciences Ethics Committee (Reference No. 559, Date: 11 March 2014).

### 2.2. Gait Analysis

Patients walked barefoot along a level walkway at a self-selected, habitual speed, and data for an average of three gait trials were collected for each patient and used for subsequent analysis. Prior to these measurements, we recorded the following patient data: height, weight, leg length (anterior superior iliac spine to medial malleolus), anterior superior iliac spine width, knee joint width, and ankle joint width. Kinematic data were obtained at 200 Hz using an eight-camera motion analysis system (Oxford Metrics Group, Vicon Nexus, Oxford, UK). For kinetics data, two floor-mounted force plates (Kistler Instruments, Winterthur, Switzerland) were used to obtain the ground reaction forces at a rate of 1200 Hz, and the data were synchronized with the motion capture data. According to a lower extremity model of the Plug-In-Gait^®^ marker set (Oxford Metrics Group) [11], which is a widely used standardized marker arrangement for three-dimensional motion analysis, 9.5-mm-diameter reflective markers were placed directly over the following bilateral anatomical landmarks: anterior and posterior superior iliac spines, lateral thighs, lateral femoral epicondyles, lateral calves, lateral malleoli, calcanei, and the tops of the feet at the base of the second metatarsals. After attaching the reflective markers, we asked each patient to stand barefoot for a single static calibration in the standing position, prior to the gait analysis. Patients were then instructed to step on a floor-mounted force plate using the lower limb targeted for measurement and were allowed to perform several preparation trials.

### 2.3. Data Analysis

After the walking measurements, we determined the foot-strike and toe-off from the ground reaction force data and identified the corresponding frame number in the data. The gait parameters during the stance phase, determined from the force plate data, were normalized to the values at 100% during the stance phase (foot-strike to toe-off = 100%) using spline interpolation. The stance phase of the gait was defined as five periods [12]: initial contact (0% of the stance: IC), loading response (16% of the stance: LR), mid-stance (50% of the stance: MS), terminal stance (83% of the stance: TS), and preswing (100% of the stance: PS). The markers and joint angles acquired from the Plug-In-Gait^®^ model were low-pass filtered at 6 Hz, and the ground reaction forces were filtered at 30 Hz using a second-order, dual-pass Butterworth filter. We modeled the body as a three-dimensional rigid link between the foot, shin, and thigh, and computed the internal moments of the knee joint using inverse dynamics techniques and formulas, as described previously [13]. Data for changes in bilateral knee joint angles in three dimensions during the stance phase and bilateral knee sagittal and frontal moments exerted during early and late stance phases were extracted from kinematics and kinetics analyses. Following patients’ interviews with the orthopedic surgeon, the side with strong knee pain causing limitations in patients’ activities of daily living was the planned operation side, while the other side served as the control side. We then compared side-to-side differences.

### 2.4. Statistical Analysis

To compare the side-to-side differences in bilateral KOA, we used the paired t-test to evaluate NRS, FTA and ROM (Table 1). In addition, the knee joint angles in three dimensions and the moments of the sagittal and frontal planes in each period during the stance phase were analyzed in the same way. The *p*-values less than 0.05 were considered statistically significant. All statistical analyses were performed using SPSS software ver. 23.0 (SPSS Inc., Armonk, NY, USA).

## 3. Results

### 3.1. Knee Joint Kinematics during the Stance Phase

The angle of the knee joint and the amount of change in every phase during the stance phase of walking are shown in Table 2 and Table 3. None of the three-dimensional joint movements in the knee joint were significantly different for all phases between KOAs and KOAw.

### 3.2. Knee Joint Moments during the Stance Phase

Knee extensor moment in the early stance phase in KOAs was significantly smaller than that in KOAw (*p =* 0.015, effect size: η_p_^2^ = 0.261, power: d = 0.714). In the late stance phase, knee extensor moment did not differ significantly between KOAs and KOAw (Table 4). In addition, KOAs and KOAw both showed large knee abductor moments during the early and late stance phases, but we found no significant difference between KOAs and KOAw (Table 4).

## 4. Discussion

The results of this study supported part of our hypothesis. There was no difference in knee joint movement, but internal knee extensor moment on the strong-pain side was significantly decreased compared with the weak-pain side.

Although it is widely known that quadriceps muscle weakness occurs in severe KOA [14,15], few reports discuss the side-to-side differences in severe KOA. Knee extensor moment during the walking stance phase is exerted in the initial stance phase. In severe KOA, exertion of knee extensor moment is smaller than that of healthy people because of pain and joint dysfunction [13]. It is thought that severe KOA reduces walking speed more than in healthy people, and soft landing on the floor reduces the exertion of knee extensor moment [16,17]. In this study, severe KOA had the same level of severity on both sides, and the exertion of the knee extensor moment was smaller for KOAs (the knee with strong pain). We consider that the exertion of the knee extensor moment decreased to avoid the pain caused by impact on the floor in KOAs. Decreased knee extensor moment secondary to pain avoidance is a factor causing functional decline secondary to quadriceps muscle atrophy, which increasingly worsens symptoms in the KOAs because of the limited range of motion and decreased muscle function.

Generally, the normal knee joint was slightly flexed from IC to LR and then extended from LR to MS [12], in our study, but our results showed that although KOAw is slightly extended in severe KOA, the KOAs remains in flexion from LR to MS. In MS, this mechanism acts to reduce the mechanical load by extending the knee joint, but a kinematic change secondary to failure of the knee joint structure in severe KOA increases the load on the joint and exacerbates pain [4,18]. Furthermore, varus thrust occurs, and the varus angle increases from IC to LR secondary to varus deformation, which increases the mechanical load on the medial side of the knee joint [19]. Knee joint motion deviated from normal in the sagittal and frontal planes concentrates the mechanical load locally on the joint plane in the initial stance phase during walking, and may promote structural failure of the knee joint. In this study, differences in the magnitude of pain in the left and right knee joints were not significant regarding kinematic changes, but deviations from normal knee joint movement in the walking stance phase reduced mobility, and we consider that this concentrates the dynamic load on the joint surface and is markedly involved in functional deterioration of the knee joint.

This study has limitations. The number of patients were not sufficient to generalize the results for all patients with severe KOA. Based on the results of this study, it is necessary to calculate the required number of subjects and to conduct a blinded prospective study in the future to generalize the results.

With bilateral severe KOA with similar side-to-side severity, the magnitude of the pain may cause dysfunction in the knee joint during the walking stance phase. It has been reported that pain affects physical function, especially walking [20]. In this study, all subjects had more pain in the surgical side. It was suggested that in the knee joint with strong pain it is difficult to exert the internal knee extensor moment and the knee joint function is reduced.

## 5. Conclusions

According to our results, with bilateral severe KOA, although there was no difference in joint motion when comparing sides, knee extensor moment on the side with strong pain was reduced and led to knee joint dysfunction. In patients with bilateral severe KOA, it was suggested that the magnitude of the pain associated with structural failure of the knee joint contributed to the decreased knee joint function.

## Figures and Tables

**Table 1:** Patients’ characteristics. (Average ± SD).

	KOAs	KOAw	*p*-Value
Subjects	11 (male = 2, female = 9)		
Age (years)	73.1 ± 6.5		
Height (m)	1.50 m ± 0.08 m		
Weight (kg)	64.1 kg ± 9.3 kg		
BMI (kg/m^2^)	28.7 kg/m^2^ ± 5.6 kg/m^2^		
Femoro–Tibial Angle (FTA)	190.8° ± 6.4°	190.5° ± 6.2°	*p* = 0.893
Range of Motion (ROM)			
Knee Flexion	115.5° ± 15.9°	115.5° ± 17.2°	*p* = 0.949
Knee Extension	−8.2° ± 7.2°	−10.0° ± 6.7°	*p* = 0.546
Pain (Numerical Rating Scale: NRS)	8.0 ± 1.9	6.9 ± 2.1	*p* < 0.001

KOAs, strong painful knee; KOAw, weak painful knee; BMI, body mass index.

**Table 2:** Knee joint kinematics in each phase during the stance phase (average ± SD).

Stance Phase	KOAs	KOAw	*p-*Value
	Flexion-extension		
IC	8.9° ± 13.0°	8.4° ± 10.6°	*p* = 0.926
LR	11.7° ± 14.6°	13.2° ± 12.9°	*p =* 0.797
MS	13.1° ± 13.3°	12.0° ± 12.0°	*p =* 0.846
TS	16.7° ± 13.5°	13.6° ± 9.5°	*p =* 0.541
PS	32.4° ±10.7°	29.4° ± 13.3°	*p =* 0.575
	Adduction-abduction		
IC	11.9° ± 5.3°	13.5° ± 5.9°	*p =* 0.507
LR	14.8° ± 6.5°	16.8° ± 6.8°	*p =* 0.509
MS	13.4° ± 7.4°	14.7° ± 7.8°	*p =* 0.692
TS	11.4° ± 6.7°	14.7° ± 7.3°	*p =* 0.281
PS	6.0° ± 7.8°	14.0° ± 11.8°	*p =* 0.078
	External-internal rotation		
IC	10.4° ± 9.5°	10.9° ± 10.6°	*p =* 0.900
LR	7.9° ± 10.0°	7.8° ± 12.0°	*p =* 0.986
MS	3.7° ± 9.6°	5.5° ± 10.6°	*p =* 0.676
TS	1.2° ± 10.2°	3.1° ± 10.7°	*p =* 0.687
PS	0.2° ± 10.3°	1.7° ± 12.0°	*p =* 0.757

KOAs, strong painful knee; KOAw, weak painful knee; IC, initial contact (0% of the stance); LR, loading response (16% of the stance); MS, mid-stance (50% of the stance); TS, terminal stance (83% of the stance); PS, preswing (100% of the stance). Flexion, adduction and external rotation show positive.

**Table 3:** Changes in each phase during the stance phase. (Average ± SD).

Stance Phase	KOAs	KOAw	*p-*Value
	Flexion-extension		
IC–LR	2.8° ± 3.1°	4.8° ± 4.5°	*p =* 0.236
LR–MS	1.4° ± 3.8°	−1.2° ± 4.5°	*p =* 0.159
MS–TS	3.6° ± 4.1°	1.6° ± 5.9°	*p =* 0.359
TS–PS	15.7° ± 8.2°	15.9° ± 11.0°	*p =* 0.970
	Adduction-abduction		
IC–LR	2.9° ± 2.2°	3.2° ± 2.7°	*p =* 0.776
LR–MS	−1.5° ± 1.8°	−2.1° ± 1.9°	*p =* 0.459
MS–TS	−2.0° ± 1.8°	0.1° ± 2.6°	*p =* 0.051
TS–PS	−5.3° ± 6.1°	−0.7° ± 7.6°	*p =* 0.131
	External-internal rotation		
IC–LR	−2.5° ± 3.1°	−3.2° ± 3.4°	*p =* 0.654
LR–MS	−4.2° ± 2.3°	−2.3° ± 2.5°	*p =* 0.077
MS–TS	−2.4° ± 3.5°	−2.4° ± 3.3°	*p =* 0.998
TS–PS	−1.1° ± 2.5°	−1.4° ± 2.9°	*p =* 0.782

KOAs, strong painful knee; KOAw, weak painful knee; IC, initial contact (0% of the stance); LR, loading response (16% of the stance); MS, mid-stance (50% of the stance); TS, terminal stance (83% of the stance); PS, preswing (100% of the stance). Flexion, adduction and external rotation show positive. Extension, abduction and internal rotation show negative.

**Table 4:** Knee joint moments during the stance phase (average ± SD).

	KOAs	KOAw	*p-*Value
Knee extensor moment at early stance phase	−0.07 Nm/kg ± 0.35 Nm/kg	0.30 Nm/kg ± 0.29 Nm/kg	*p =* 0.015
Knee extensor moment at late stance phase	0.38 Nm/kg ± 0.27 Nm/kg	0.54 Nm/kg ± 0.43 Nm/kg	*p =* 0.314
Knee abductor moment at early stance phase	1.10 Nm/kg ± 0.56 Nm/kg	1.37 Nm/kg ± 0.43 Nm/kg	*p =* 0.210
Knee abductor moment at late stance phase	1.10 Nm/kg ± 0.49 Nm/kg	1.47 Nm/kg ± 0.53 Nm/kg	*p =* 0.107

KOAs, strong painful knee; KOAw, weak painful knee.
